# Induction of Olig2^+^ Precursors by FGF Involves BMP Signalling Blockade at the Smad Level

**DOI:** 10.1371/journal.pone.0002863

**Published:** 2008-08-06

**Authors:** Bilada Bilican, Christelle Fiore-Heriche, Alastair Compston, Nicholas D. Allen, Siddharthan Chandran

**Affiliations:** 1 Department of Clinical Neurosciences and Centre for Brain Repair, University of Cambridge, Cambridge, United Kingdom; 2 Department of Oncology Hutchison/MRC Research Centre, University of Cambridge, Cambridge, United Kingdom; 3 School of Biosciences, Cardiff University, Cardiff, United Kingdom; Columbia University, United States of America

## Abstract

During normal development oligodendrocyte precursors (OPCs) are generated in the ventral spinal cord in response to Sonic hedgehog (Shh) signalling. There is also a second, late wave of oligodendrogenesis in the dorsal spinal cord independent of Shh activity. Two signalling pathways, controlled by bone morphogenetic protein and fibroblast growth factor (FGF), are active players in dorsal spinal cord specification. In particular, BMP signalling from the roof plate has a crucial role in setting up dorsal neural identity and its inhibition is sufficient to generate OPCs both *in vitro* and *in vivo*. In contrast, FGF signalling can induce OPC production from dorsal spinal cord cultures *in vitro*. In this study, we examined the cross-talk between mitogen-activated protein kinase (MAPK) and BMP signalling in embryonic dorsal spinal cord cultures at the SMAD1/5/8 (SMAD1) transcription factor level, the main effectors of BMP activity. We have previously shown that FGF2 treatment of neural precursor cells (NPCs) derived from rat E14 dorsal spinal cord is sufficient to generate OPCs *in vitro*. Utilising the same system, we now show that FGF prevents BMP-induced nuclear localisation of SMAD1-phosphorylated at the C-terminus (C-term-pSMAD1). This nuclear exclusion of C-term-pSMAD1 is dependent on MAPK activity and correlates with OLIG2 upregulation, the obligate transcription factor for oligodendrogenesis. Furthermore, inhibition of the MAPK pathway abolishes OLIG2 expression. We also show that SMAD4, which acts as a common partner for receptor-regulated Smads including SMAD1, associates with a Smad binding site in the *Olig2* promoter and dissociates from it upon differentiation. Taken together, these results suggest that FGF can promote OPC generation from embryonic NPCs by counteracting BMP signalling at the Smad1 transcription factor level and that Smad-containing transcriptional complexes may be involved in direct regulation of the *Olig2* promoter.

## Introduction

The vertebrate brain is composed of a variety of neural cell types which are generated from neural precursor cells (NPCs) that have the potential to differentiate into neurons, astrocytes, and oligodendrocytes [1]. Differentiation of NPCs into neurons and glia occurs in temporally distinct waves with neurogenesis preceding gliogenesis [2]. The origin of oligodendrocytes, the myelinating cells of the vertebrate central nervous system (CNS), has been studied extensively in the developing spinal cord and two distinct phases of oligodendrogenesis have been established. OPCs first originate from the motor neuron progenitor (pMN) domain of the ventral neural tube under the influence of Shh signalling from the notochord and floorplate [3], [4]. In addition Shh has been implicated in the proliferation, maintenance and migration of adult neural precursors and their derivatives [5]–[8]. The ventrally-derived OPCs migrate laterally and dorsally, populating the CNS before maturing into myelin-forming oligodendrocytes. A second phase of oligodendrogenesis takes place in the embryonic dorsal spinal cord (DSC) which is independent of Shh signalling [9], [10]. Using *in vitro* systems, it has been shown that Shh signalling is not essential for oligodendrogenesis as NPCs isolated from the embryonic spinal cord can generate oligodendrocytes in the presence of cyclopamine-KAAD (a potent blocker of hh signalling) [11], [12]. In addition, oligodendrocytes may still be produced by NPCs isolated from Shh functional knockout mice [12]. Later studies by Cai et al. (2005) and Vallstedt et al. (2005) confirmed that *in vivo,* oligodendrocytes originate in the developing DSC as well as in the ventral pMN domain.

The BMPs are members of the TGF-β family and are known inhibitors of neuronal differentiation [13]. In addition, BMP signalling is known to inhibit Shh-induced oligodendrogenesis and it has been shown that inhibition of BMP signalling is sufficient to induce oligodendrocyte generation both *in vitro* and *in vivo* [9], [14], [15]. BMPs are secreted signalling proteins that bind to cell-surface serine/threonine kinase receptors [16]. Activated receptor kinases in turn relay this signal to the nucleus via activation of Smad transcription factors, which is achieved by phosphorylation of the C-terminal SXS motif [17]. There are five mammalian receptor-regulated Smads (R-Smads) (Smads 1–3, 5 and 8) that serve as substrates for the TGF-β receptor family. Smads 1, 5, and 8 are targets for BMP and anti-Mullerian receptors, whereas Smads 2 and 3 are regulated by TGF-β, Nodal and activin receptors [17], [18]. In addition to receptor-mediated activation the formation of transcriptionally active Smad complexes also requires dimerisation of R-Smads with SMAD4, also referred as Co-Smad [17]. R-Smad/SMAD4 oligomers form the core of diverse multi-subunit transcriptional regulation complexes, which include other DNA sequence-specific binding proteins and co-factors. Altogether the combination of these factors determine target specificity, leading to activation or repression of certain genes [17].

Two bHLH transcription factors, Olig1 and Olig2, are also known to regulate oligodendrocyte specification in the developing nervous system [19]–[23]. Specifically, Olig2 is an obligate factor for oligodendrogenesis in the developing spinal cord as the disruption of Olig2 alone results in complete elimination of oligodendrocytes [19], [20]. Regulation of *Olig1* and *Olig2* expression presents a key step in oligodendrocyte differentiation and two signalling pathways, MAPK and BMP, are known to play opposing roles in this process. BMP mediated signalling blocks dorsal oligodendrocyte specification *in vitro*, and their generation is promoted when BMP signalling is inhibited [12]. Indeed, production of OLIG2^+^ dorsally-derived OPCs which arise later than their ventral counterparts correlates with a decrease in BMP signalling [9], [10]. In contrast, FGF signalling has been shown to promote Olig2 expression and oligodendrocyte differentiation *in vitro* [11], [12] via MAPK signalling, including in dorsal explants [10]. Furthermore, exposure of dorsal PAX7^+^ cells to a FGF receptor antagonist abolishes any OLIG2 expression [9] indicating that FGF activity is required for oligodendrogenesis in the DSC.

BMP-regulated Smads lie at the crossroads of the antagonistic signalling interplay between MAPK and BMP pathways. BMP receptor kinases relay signals through C-terminal phosphorylation and nuclear translocation of the transcription factor SMAD1, whereas MAPKs catalyse inhibitory phosphorylation in the SMAD1 linker region that results in the cytoplasmic sequestration of SMAD1 [17], [18], [24], [25]. Recently, the regulation of SMAD1 activity has been elucidated in detail revealing interplay of MAPK, Smurf1 and nucleoporin interactions that control the cytoplasmic sequestration and turnover of SMAD1 protein [26].

Here, we show that BMP signalling pathway is active in rat embryonic DSC precursor cultures and that the MAPK pathway is responsive to FGF2 treatment. We also show that BMP-activated SMAD1, that is C-term-pSMAD1, is sequestered in the cytoplasm in a MAPK-dependent manner accompanied with increased OLIG2 expression suggesting that FGF2 can potentially antagonise BMP signalling in the embryonic DSC during oligodendrogenesis. SMAD4, which is also referred as Co-Smad due to its dimerisation with R-Smads, associates with the *Olig2* promoter in undifferentiated cells and this interaction is lost upon induction of *Olig2* transcription suggesting that BMP signalling may directly regulate *Olig2* expression.

## Results

### Smad expression and MAPK signalling activity in DSC precursor cultures

We and others have shown that FGF2 can induce Shh-independent production of oligodendrocytes by dissociated NPCs and in dorsal explants [10], [11]. FGFs, through binding to their cognate receptors, activate several signal transduction cascades including the extracellular signal-regulated protein kinase (ERK) cascade which is part of the canonical MAPK pathway [27]. MAPK pathway activators counterbalance BMP action during neurogenesis, bone formation, and other aspects of vertebrate development. BMP receptors signal through C-terminal phosphorylation and nuclear translocation of the transcription factor SMAD1, whereas MAPK catalyses inhibitory phosphorylation in the SMAD1 linker region. To determine if such a mechanism of action is responsible for FGF2 dependent oligodendrogenesis from rodent dorsal cord, we established primary cultures of DSC NPCs from E14 embryos, at which point there are no constitutive dorsal OPCs [12], [28].

**Figure 1:**
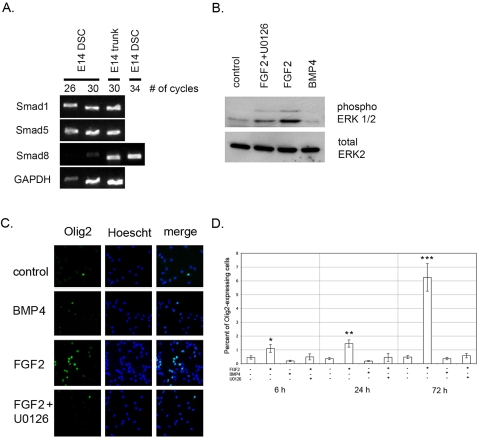
Smad expression, MAPK signalling analysis and quantification of OLIG2 expressing cells in DSC neural precursor cell cultures. A. Semi-quantitative RT-PCR analysis of *Smad1*, *5*, and *8* expression in primary E14 rat dorsal spinal cord cultures. Rat E14 trunk cDNA was used as positive control. B. Western blot analysis of extracts from different DSC culture conditions using anti-phospho ERK1/2 polyclonal antibody to assess MAPK activity. Anti-ERK2 antibody was used as a loading control. C. Primary cultures of E14 dorsal neural precursor cells were treated with FGF2, BMP4 or FGF2+U0126 for 6, 24 or 72 hours and stained for OLIG2 expression. Pictographs showing representative fields for different culture conditions after 72 hours of treatment. D. Cells expressing OLIG2 were counted and represented as percent of the total cell number. Data represent mean +/− SEM. FGF2 treatment 72 h time point is statistically different from 6 and 24 h (ANOVA with Tukey HSD test, p<0.01). FGF2 treatment is also statistically different at each time point compared to other conditions (*,p<0.05; **, p<0.0001; *** p<0.0001; ANOVA with Tukey HSD).

First, we established the expression pattern of the downstream effectors of BMP signalling in our culture system. Semi-quantitative RT-PCR expression analysis of *Smad 1*, *5*, and *8* revealed that *Smad1* and *Smad5* were expressed at high levels whereas *Smad8* transcript levels were much lower (Figure 1A, compare cycle numbers 26 and 30 for *Gapdh*, *Smad1/5/8*). All three transcripts were readily detected in total E14 trunk cDNA at cycle 30. These results showed that there is preferential expression of *Smad1* and *Smad5* in the E14 rat dorsal spinal cord.

We next examined the MAPK pathway activity in different DSC culture conditions. Primary DSC precursor cells were cultured for 12 hours after isolation and were subsequently treated for 6 hours with FGF2, FGF2+U0126 (an inhibitor of MAPK signalling), BMP4 or DMSO (control). Western blotting using an antibody to phospho-ERK1/2, specific for the active form of the enzyme, enabling assessment of MAPK activity, was next undertaken. A low level of MAPK activity in DSC cells was detected under control and BMP4-treated conditions (Figure 1B). Treatment of DSC precursor cultures with FGF2, however, resulted in robust activation of MAPK signalling pathway as judged by increased levels of phospho-ERK1/2. This effect was reduced in the presence of U0126 (Figure 1B). These results are consistent with the absence of strong, sustained MAPK activity in dissociated DSC cultures and further that MAPK signal transduction pathway is responsive to stimulation by FGF2 treatment.

### FGF2 induces OLIG2^+^ cells

Following confirmation of very low numbers of OLIG2^+^ cells (Figure 1C and 1D, control 6h 0.45±0.12%) in primary DSC cultures, we next examined whether FGF2 treatment results in upregulation of OLIG2 expression. Addition of BMP4 did not significantly influence OLIG2^+^ cells (Fig 1C and 1D). However, treatment with FGF2 resulted in a substantial increase in numbers of OLIG2^+^ cells; 1.46±0.26% and 6.25±1.00% after 24 and 72 hours, respectively and was significant compared to control and BMP4 treated cultures (Fig 1C and 1D). Co-treatment with MAPK inhibitor U0126 and FGF2 resulted in a negligible increase in OLIG2^+^ cells indicating FGF2-induced OLIG2 expression is MAPK signalling dependent (Figure 1D). To investigate whether increased BMP signalling can antagonise induction of OLIG2 expression by FGF2, primary DSC cultures were co-treated with FGF2 and BMP4 for 72 hours. The number of OLIG2^+^ cells was reduced by 60.75% compared to FGF2 only (p<0.01) suggesting that BMP signalling can directly compete with the effects of FGF2.

### FGF2 regulates sub-cellular localisation of SMAD1

**Figure 2:**
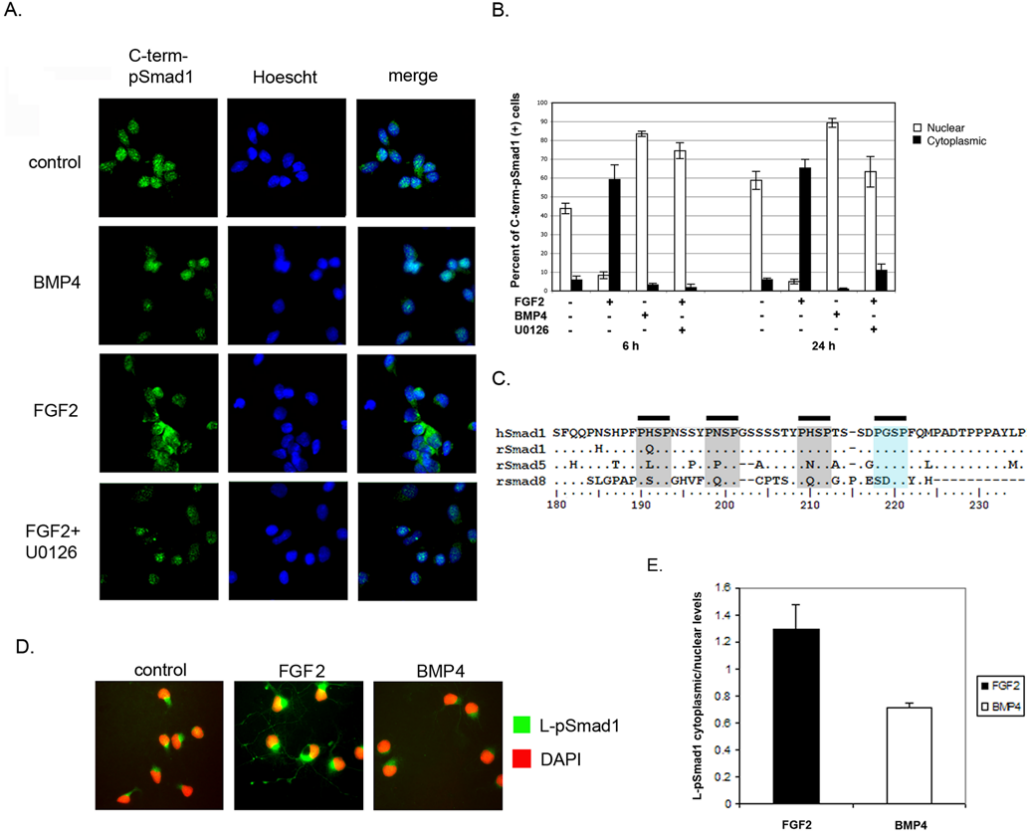
FGF2 regulates sub-cellular localisation of SMAD1. A. Primary cultures of E14 dorsal neural precursor cells were treated with FGF2, BMP4 or FGF2+U0126 for 6 hours or 24 hours and labelled for C-term-pSMAD1. Representative fields from 6 hours treatment is shown. B. Cells showing an exclusive nuclear or an exclusive cytoplasmic localisation were counted and are represented as a percent of total C-term-pSMAD1 expressing cells. Data represent mean +/− SEM. FGF2 treatment at each time point is statistically different from the three other groups (ANOVA and t-Test with Bonferonni, p<0.001). C. Alignment of the linker regions of receptor-regulated Smads and identification of MAPK phosphorylation sites. Conserved phosphorylation motifs between human SMAD1 and rat SMADs are highlighted in grey; green marking the peptide L-pSMAD1 antibody is raised against. D. Primary cultures of E14 dorsal neural precursor cells were treated with FGF2, or BMP4 for 6 hours and stained for L-pSMAD1. Representative fields are shown. E. Comparison of L-pSMAD1 cytoplasmic/nuclear signal levels between FGF2 and BMP4 treated samples. Data represent mean +/− SEM (p<0.05, paired T-test).

Receptor-mediated phosphorylation of Smad factors at the C-terminus (C-term-pSmad) results in their nuclear accumulation, ultimately converting the BMP signal to a transcriptional readout. To analyse the subcellular localisation of C-term-pSMAD1 in response to different growth conditions, dissociated cells were grown in the presence or absence of FGF2 (as shown in Figure 1B) for different periods of time and the localisation of C-term-pSMAD1 examined using immunocytochemistry. Under control conditions, when BMP signalling was active, C-term-pSMAD1 was predominantly nuclear (Figure 2A). Addition of BMP4 resulted in a near exclusive nuclear localisation (Figure 2A). FGF2 treatment for 6 hours resulted in nuclear exclusion of C-term-pSMAD1. To test if the effect of FGF2 on C-term-pSMAD1 localisation was due to MAPK activity, the specific MEK inhibitor U0126 was added to DSC cultures. Addition of U0126 together with FGF2 abolished the cytoplasmic sequestration/nuclear exclusion of C-term-pSMAD1 (Figure 2A and 2B) showing that C-term-pSMAD1 requires MAPK signalling for its nucleo-cytoplasmic shuttling.

It has previously been shown that deactivation of SMAD1 requires phosphorylation at the linker region by the MAPK pathway [24], [25] and that this linker phosphorylation event can result in cytoplasmic retention of SMAD1 [25]. There are four MAPK phosphorylation sites conserved between linker regions of Smad1, 5 and 8 in humans [26]. To investigate if there was a change in linker-phosphorylated SMAD1 (L-pSMAD1) levels in response to FGF2 treatment, we repeated the earlier experiment with analysis of L-pSMAD1 expression. The polyclonal antibody used to detect L-pSMAD1 was raised against the fourth MAPK phosphorylation site in the linker region of hSMAD1 (Figure 2C) [29]. Amino acid sequence alignment between the linker regions of human SMAD1 and rat SMAD1, 5 and 8 proteins showed that the fourth MAPK phosphorylation site, and the antigen used for immunisation, is identical between human and rat Smad1 orthologues (Figure 2C, [29]). Sequence alignment also revealed that the fourth phosphorylation site was conserved in Smad5 but was not present in Smad8 suggesting that L-pSMAD1 antibody is likely to recognise both linker-phosphorylated rat Smad1 and Smad5 paralogues. Upon FGF2 treatment, cytoplasmic L-pSMAD1 levels increased and was clearly visible in cytoplasmic processes of some cells (Figure 2D, compare control and FGF2 treated). Furthermore, BMP4 treatment did not effect the basal level of L-pSMAD1 as detected by immunofluorescence (Figure 2D) and the quantification of cytoplasmic/nuclear L-pSMAD1 signal ratios showed that FGF2 treatment resulted in 73% increase in L-pSMAD1 levels in the cytoplasm compared to BMP4 (Figure 2E, relative cytoplasmic/nuclear signal intensity: FGF2 1.335 +/− 0.156, BMP4 0.71 +/− 0.23, p<0.05, paired T-test). Taken together, these data suggest that FGF2 treatment can antagonise BMP signalling by regulating the subcellular localisation of SMAD1 via the MAPK pathway in dissociated rat embryonic DSC cultures and that OLIG2 expression correlates with nuclear exclusion of C-term-pSMAD1. Since Shh and FGF2 signaling provide independent mechanisms of inducing Olig2 expression and oligodendrogenesis [14], [15] one possibility would be for activated-SMAD subcellular localisation to provide a point of cross-talk between the two pathways. However, analysis of Shh treated rat DSC cells did not result in any significant subcellular localisation change of C-term-pSMAD1 (result not shown) suggesting that SMAD signalling does not provide a point of cross-talk between the two pathways.

### SMAD4 associates with Smad binding sites in *Olig2* promoter

**Figure 3:**
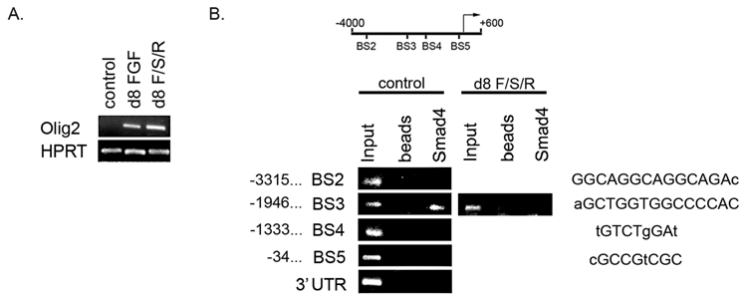
SMAD4, co-partner for all Receptor-Smads, binds to *Olig2* promoter. A. Semi-quantitative RT-PCR analysis of *Olig2* expression in undifferentiated (control) and neuralised (FGF2 only and FGF2/Shh/Retinoic Acid (F/S/R)) W9.5 mES cells. mES cells neuralised in chemically defined medium (CDM) for 4 days were further differentiated for 4 days in CDM+FGF2 (d8 FGF) or CDM+F/S/R (d8 F/S/R). *Olig2* transcription is not detected in undifferentiated mES cells but is strongly induced upon 4 day differentiation in FGF2/Shh/Retinoic Acid containing medium. B. Schematic representation of putative Smad binding sites (BS) targeted for analysis in 4 kb promoter region of *Olig2*. PCR products identified by agarose gel electrophoresis after chromatin immunoprecipitation from W9.5 mES cells with Smad4 antibody. Smad4 binding was detected at BS3 only. No Smad4 binding is detected using beads only or at 3′ UTR of *Olig2*. Smad4 binding at BS3 site is lost upon differentiation. Nucleotides represented in uppercase are exact matches to previously described consensus binding sequences at respective Smad sites in the *Olig2* promoter. The positions of the binding sites are shown relative to the transcription start site.

Olig2 is a bHLH transcription factor with a critical role in both motor neuron and oligodendrocyte specification. Its expression is regulated both temporally and spatially during neural development [19], [30], [31] . However, the promoter of the *Olig2* gene is not described in the literature and there is limited information on trans-acting factors that regulate its expression. Recently, using mouse embryonic stem (mES) cells as a model for analysing neural gene transcription it was shown that *Olig2* has a complex promoter structure with a 2 kb proximal region that contributes significantly to promoter activity [32]. In mES cells BMP4 acts in combination with LIF to sustain self-renewal and preserve multilineage differentiation potential [33]. To investigate if BMP4 signalling can potentially regulate *Olig2* expression via Smads undifferentiated mES cells were studied. Undifferentiated mES cells do not express *Olig2* as determined by semi-quantitative RT-PCR (Figure 3A) but can be induced, in defined conditions, upon addition of FGF2 or with combinatorial application of FGF2, retinoic acid and Shh during directed differentiation of mES cells to motor neurons (Figure 3A) [34], [35]. Putative Smad binding sites were determined in the 4 kb upstream sequence from the transcription start site of mouse *Olig2* using MatInspector and rVista2 [36], [37]. Four sites which are conserved between mouse and rat were chosen to be studied for *in vivo* SMAD4 association using chromatin immunoprecipitation. SMAD4 was chosen on the basis that all Smad transcriptional complexes identified to date contain SMAD4 [17]. Our results indicate that Smad4 binds specifically to one of these promoter elements in undifferentiated mES cells (Figure 3B, BS3). Interestingly, upon directed differentiation of mES cells towards motor neurons, with concomitant high *Olig2* expression (Figure 3A, d8 F/R/S lane), SMAD4 occupancy at BS3 is no longer detectable with ChIP (Figure 3B) suggesting that SMAD4 containing complexes may directly regulate the *Olig2* promoter.

## Discussion

Fate specification and differentiation requires the interplay of multiple developmental signals. We provide evidence using a well characterised developmental model that dorsal cord induction of Olig2 is dependent on MAPK activity that antagonises BMP mediated signalling resulting in cytoplasmic sequestering of C-term-pSMAD1. Furthermore, we provide evidence that SMAD4, a common partner for receptor-regulated Smads including SMAD1, associates with a Smad binding site in the *Olig2* promoter with subsequent dissociation upon differentiation.

In general, it is believed that BMP signalling prevents neuronal differentiation. However, in dissociated *Xenopus* embryonic ectodermal cells sustained MAPK activity causes neuronal differentiation despite the presence of BMP signalling, showing that MAPK signalling can counteract the BMP pathway [29]. Our results show that BMP signalling is intact in dissociated rat embryonic DSC cultures, where the transcription factors *Smad1* and *Smad5* are transcribed highly and BMP receptor kinase-activated SMAD1, C-term-pSMAD1, is present. However, there is no strong and sustained MAPK activity in embryonic DSC cultures, demonstrated by very low levels of activated ERK1/2 in control conditions, in contrast to *Xenopus* embryonic ectodermal cells [29]. Nevertheless, treatment with FGF2 resulted in activation of the MAPK pathway revealing that DSC derived NPCs are responsive to MAPK activators. Further experiments are needed to determine which FGF receptors are present on precursor cells.

Developmental origin of oligodendrocytes has been an area of much study. In addition to early restricted ventral foci of OPCs, recent studies have determined a second dorsal wave of oligodendrogenesis both in the spinal cord and forebrain [9], [10], [38]. The early ventral phase of spinal cord oligodendrogenesis originates in the pMN domain. The second dorsal derived wave, in contrast to oligodendrocyte generation in the ventral neural tube, is independent of Shh-signalling [10]–[12]. It is suggested that oligodendrogenesis in the developing dorsal neural tube results from integration of FGF signalling and decreasing BMP action in the region [9], [10]. Recent work on mouse mesencyhmal progenitor cells and neuroectodermal explants in *Xenopus* has elucidated, at the molecular level, how MAPK signalling can oppose BMP activity via the SMAD1 transcription factor. Signalling events triggered by different factors such as FGF8, IGF2, JNK and p38 can all lead to linker-phosphorylation of SMAD1 via MAPK pathway activity [24]–[26]. This in turn leads to degradation of SMAD1 via the proteasome [26] and/or cytoplasmic retention of the protein [26], [39]. The nuclear exclusion and degradation of SMAD1 are not mutually exclusive events as the binding of SMURF1 to SMAD1, which leads to SMAD1 polyubiquitination also inhibits the interaction of SMAD1 with the nuclear pore complex [26]. Our results suggest that FGF signalling can directly counteract BMP action in DSC precursor cells by regulating the subcellular localisation of BMP receptor-kinase activated SMAD1 (C-term-pSMAD1). The transcriptionally active form of SMAD1, C-term-pSMAD1, is predominantly nuclear under control conditions where there is ongoing BMP signalling. Receptor-regulated Smads undergo continuous nucleo-cytoplasmic shuttling enabling them to respond to changing stimuli [17]. We report that treatment of DSC precursor cultures with FGF2 activates the MAPK pathway and results in rapid cytoplasmic sequestration of C-term-pSMAD1, thereby inhibiting BMP signalling. Concomitantly, linker-phosphorylated SMAD1 (L-pSMAD1) levels were observed to increase in the cytoplasm. Furthermore, the basal level of cytoplasmic L-pSMAD1 signal observed in cells under BMP and control conditions suggest a continuous turn-over of the Smad1 protein in DSC neural precursor cells.

Oligodendrogenesis in the CNS is regulated by two bHLH transcription factors, Olig1 and Olig2 [19]–[21], [30]. Treatment of DSC precursor cells with FGF2 resulted in significant and rapid increase in numbers of OLIG2^+^ cells that correlated with the de-activation/cytoplasmic sequestration of SMAD1. One outstanding question is whether the *Olig2* promoter is regulated by Smads directly. Recent developments of detailed differentiation protocols for neural cells from mES cells provide a valuable platform to study the regulation of neural gene transcription. ES cell differentiation *in vitro* is thought to recapitulate *in vivo* developmental programmes [40]–[43]. In mES cells BMP4 acts in combination with LIF to sustain self-renewal and preserve multilineage differentiation, chimera colonisation, and germline transmission properties. A critical contribution of BMP4 is to induce expression of *Id* genes via the Smad pathway, which suppresses differentiation to neural lineages [33]. Our ChIP results show that SMAD4, also known as Co-Smad, can associate with the *Olig2* promoter in undifferentiated mES cells. Moreover, upon induction of *Olig2* expression in mES cells during motor neuron differentiation SMAD4 is displaced from the BS3 site on the *Olig2* promoter suggesting that BMP signalling may directly regulate the expression of *Olig2*. Recently it was shown that the conditional deletion of *Smad4* in the adult mouse subependymal zone resulted in elevated expression of OLIG2, implicating SMAD4 containing complexes in the modulation of *Olig2* expression [44]. Upon activation by phosphorylation, receptor-regulated Smads (Smad1/5/8 in the case of BMP signalling) dimerise with SMAD4 and assemble multi-protein transcription regulatory complexes that target several promoters for activation or repression [17]. For instance, the transcriptional repressor Nkx3.2, which can induce chondrocyte differentiation in the presence of BMP signalling [45], forms a complex with SMAD1/SMAD4 and mSin3/HDAC co-repressor complex demonstrating that BMP-activated Smads can be involved in transcriptional repression [46]. It is thus plausible that BMP signalling assembles a SMAD-dependent transcriptional repression complex on the *Olig2* promoter and that FGF2 signalling via MAPK activity relieves this repression by sequestering SMAD1 in the cytoplasm. The presence of multiple highly homologous receptor-regulated Smads in tissues, as in the high level expression of *Smad1* and *Smad5* in DSC precursor cells, raises the possibility of functional redundancy among the transcription factor family. Further functional studies are required to investigate *Olig2* promoter structure, contribution of the putative Smad binding sites to the promoter activity along with the associated Smads and the composition of the transcriptional regulation complexes that may target these sites.

## Materials and Methods

### Primary and mES cell culture

The care and treatment of the animals used in this study was in accordance with UK Home Office and University of Cambridge regulations. Primary rat DSC cultures were performed as previously described [12]. Briefly, spinal cords were dissected out from embryonic day 14 Sprague Dawley rats (Charles River Laboratories, U.K.). Only the dorsal columns were collected. The tissue was dissociated in 0.1% trypsin (Sigma, U.K.) for 15 min at 37°C and after addition of DNase (0.001%, Sigma, U.K.) and centrifugation at 1,000 g for 3 min, cells were platted on poly-d-lysine coverslips at 50,000 cells/well in 6 wells plates and grown in Dulbecco's Modified Eagle's Medium (DMEM, Gibco, Invitrogen, U.K.) containing 2% B27 supplements (Gibco. Invitrogen, U.K.) and 1% penicillin/streptomycin and amphotericin (PSF, Sigma, U.K.) at 37°C in 5% CO_2_. When tested, growth factors were added after an overnight culture at the following concentrations: FGF2 20 ng/ml (R&D Systems Inc.), BMP4 10 ng/ml (R&D Systems Inc.) and U0126 10 µM. Cells were grown for 6 to 72 h before analysis. W9.5 mES cell line was maintained and neuralised as explained in detail in Bouhon et al., (2006). Briefly, enzymatically dissociated mES cells were resuspended in chemically defined medium (CDM) and plated at 1×10^5^ cells per ml in 10-cm bacteriological grade culture dishes. Cultures were passaged by dissociating spheres by mechanical trituration. At day 4, cultures were treated with either FGF2 (control) or FGF2/Shh/Retinoic acid (neuralisation) for 4 more days. Growth factors were used at the following concentrations: fibroblast growth factor 2 (FGF2), 20 ng/ml (R&D Systems Inc.); Sonic hedgehog N-terminal peptide (SHH-N), 250 ng/ml (R&D Systems Inc.); all-trans-retinoic acid (RA), 1 µM (Sigma-Aldrich).

### Immunocytochemistry

After 6, 24, or 72 h in culture, cells were washed with PBS and fixed 5 min with 4% paraformaldehyde. For C-term-pSMAD1 staining, cells were washed 3 times with PBS and permeabilized with ice-cold methanol for 10 min at −20°C. After blocking in 10% normal goat serum (NGS) in PBS containing 0.05% Triton X100 for 1 h, cells were incubated overnight at 4°C with anti-pSMAD1/5/8 antibody (Cell Signaling Technologies, U.S.A., Cat no: 9511S, phosphoSmad1/5/8 (Ser 463/465, Ser 426/428, Ser 463/465)) diluted 1∶100 in PBS containing 2% NGS and 0.05% Triton X100. After three washes in PBS, goat anti-rabbit antibody coupled to biotin (Molecular Probes, Invitrogen ,1∶200 in PBS) was applied for 1 h at room temperature then FITC-coupled streptavidin (Molecular Probes, Invitrogen ,1∶200 in PBS) and Hoescht (Sigma, 1∶5000 in PBS) were applied for 1 hour at room temperature. For Olig2 staining, cells were washed 3 times with PBS, blocked in PBS/10% NGS/0.05% Triton X100 for 1 h, and incubated overnight at room temperature with anti-Olig2 antibody (gift from Dr. Takeyabashi) diluted 1∶3,000 in 2%NGS/PBS/0.05% Triton X100. After three washes in PBS, goat anti-rabbit coupled to Alexa 488 (Molecular Probes, Invitrogen, 1∶500 in PBS) was applied for 1 h at room temperature together with Hoescht (Sigma, 1∶5000). L-pSMAD1 polyclonal antibody was kindly provided by Dr. De Robertis and was used at 1∶5000 following standard protocols [29]. Coverslips were mounted in Vectashield mounting medium (Vectra Laboratories, U.K.)

### Quantification and statistical analysis

Immunofluorescence was observed under a Leitz microscope at high magnification (×40) and at least 5 fields/coverslips, 2 coverslips/experiment from at least 3 different experiments (n = 5 for Olig2 quantification) were taken into pictures for quantification. For Olig2 staining, the number of positive cells as well as the total number of cells were counted. C-term-pSMAD1 staining was counted in the different cellular compartments and total cell number was assessed. The mean value of the 2 coverslips of each experiment was used to calculate the average and the standard error between the different experiments. The significance of the different treatments on C-term-pSMAD1 localisation and on Olig2 expression were assessed by one-way analysis of variance (ANOVA) followed by Tukey HSD test or t-Test with Bonferoni correction, as indicated in the text. Cytoplasmic/nuclear L-pSMAD1 levels were quantified using ImageJ software with MRI Cell Image Analyzer plugin, averaged over 4 random fields/experiment from 4 different experiments.

### Immunoblotting

Immunoblotting was carried out according to standard procedures. Protein samples were subjected to electrophoresis on denaturing 10% polyacrylamide gels, which were then transferred to PVDL membranes (Immobilon-P, Millipore) and analyzed by Western blotting. The proteins were detected with ECL reagent (GE Healthcare) in conjunction with appropriate horseradish peroxidase coupled secondary antibodies (BioRad). The antibodies used were rabbit polyclonal anti-ERK2 (Santa-Cruz SC-154) and anti-phospho p44/42 MAPK (Thr 202/Tyr204) (Cell Signaling Technologies 9101S).

### RNA Isolation and RT-PCR

Total cellular RNA was extracted from primary rat DSC cultures or undifferentiated or neuralised W9.5 mES cells using the RNeasy Mini isolation kit (Qiagen). RNase-free-DNase (New England Biolabs) treated RNA samples (2 µg) were reverse transcribed in 100 µl with random hexamers using MMLV RT (Invitrogen) according to manufacturer's protocol. PCR was conducted in 25 µl reaction volume using 2 µl cDNA synthesised as described above with BioTaq polymerase (BioLine). The primers used were: mouse hypoxanthine phosphoribosyl-transferase (mHPRT) forward:

5′-AGCTACTGTAATGATCAGTCAACG-3′;

mHPRT reverse: 5′- AGAGGTCCTTTTCACCAGCA-3′;

mOlig2 forward: 5′-GTGGCTTCAAGTCATCTTCC-3′;

mOlig2 reverse: 5′-GTAGATCTCGCTCACCAGTC-3′;

rat Smad1 forward: 5′- GGCCAGCCGCTATGAATGTGA-3′;

rSmad1 reverse: 5′-TGGGAACTCGCAGCATTCCAG-3′;

rSmad5 forward: 5′- GGCTCTGACGATTCGTATCAA-3′;

rSmad5 reverse: 5′- AGGAAACTCACAAATATCCAA-3′;

rSmad8 forward: 5′- TCCGAGTCAGACAGTCCTTAT-3′;

rSmad8 reverse: 5′- GTACAAATGCACACCCTTTCC-3′;

rGAPDH forward: 5′-TTCCAGTATGACTCTACCC-3′;

rGAPDH reverse: 5′-ATGGACTGTGGTCATGAGCCC-3′. To compensate for variable RNA and cDNA yields, the expression of HPRT was used as a control in mES experiments for which the optimal number of PCR cycles for linear amplification was determined. The amplification products were analyzed by agarose gel electrophoresis.

### Chromatin Immunoprecipitation

Chromatin immunoprecipitation was carried out with polyclonal Smad4 antibody (Santa Cruz Biotechnology, SC-7154X) using a modified protocol (PROT11) which can be found at http://www.epigenome-noe.net/researchtools/protocol.phpprotid10. Smad binding sites were identified using MatInspector and rVista2 [36], [37]. Primers were designed to encompass the selected sites (see Figure 3B for details) as follows:

BS2 forward: 5′-TAGTCCTTGCACAATTGCGT-3′;

BS2 reverse 5′-AAGCCAGAAGGACTGTAGAATGG-3′;

BS3 forward: 5′-ACCTATCTCCCGCATATTGTACC-3′;

BS3 reverse: 5′-GACCCATAACCGTTCAATTAGC-3′;

BS4 forward: 5′-ATAAATATCCCAACAAACAAACA-3′;

BS4 reverse: 5′-AATTCCTAGGTTTTCACTTCCATAA-3′;

BS5 forward: 5′-CCTCCTCCCATCCCTCCTCGC-3′;

BS5 reverse: 5′- GGTTCCGCTGGTTTTTATAGC-3′;

Olig2 3′UTR forward: 5′- ATTGGTTTCTTACCCGACTGG-3′;

Olig2 3′ UTR reverse: 5′-GGAGTCACGTGAACAAAGAGC-3′.

## References

[1] Gotz M, Huttner WB (2005). The cell biology of neurogenesis.. Nat Rev Mol Cell Biol.

[2] Richardson WD, Kessaris N, Pringle N (2006). Oligodendrocyte wars.. Nat Rev Neurosci.

[3] Pringle NP, Yu WP, Howell M, Colvin JS, Ornitz DM (2003). Fgfr3 expression by astrocytes and their precursors: evidence that astrocytes and oligodendrocytes originate in distinct neuroepithelial domains.. Development.

[4] Orentas DM, Hayes JE, Dyer KL, Miller RH (1999). Sonic hedgehog signaling is required during the appearance of spinal cord oligodendrocyte precursors.. Development.

[5] Loulier K, Ruat M, Traiffort E (2006). Increase of proliferating oligodendroglial progenitors in the adult mouse brain upon Sonic hedgehog delivery in the lateral ventricle.. J Neurochem.

[6] Bambakidis NC, Miller RH (2004). Transplantation of oligodendrocyte precursors and sonic hedgehog results in improved function and white matter sparing in the spinal cords of adult rats after contusion.. Spine J.

[7] Merchan P, Bribian A, Sanchez-Camacho C, Lezameta M, Bovolenta P (2007). Sonic hedgehog promotes the migration and proliferation of optic nerve oligodendrocyte precursors.. Mol Cell Neurosci.

[8] Palma V, Lim DA, Dahmane N, Sanchez P, Brionne TC (2005). Sonic hedgehog controls stem cell behavior in the postnatal and adult brain.. Development.

[9] Vallstedt A, Klos JM, Ericson J (2005). Multiple dorsoventral origins of oligodendrocyte generation in the spinal cord and hindbrain.. Neuron.

[10] Cai J, Qi Y, Hu X, Tan M, Liu Z (2005). Generation of oligodendrocyte precursor cells from mouse dorsal spinal cord independent of Nkx6 regulation and Shh signaling.. Neuron.

[11] Kessaris N, Jamen F, Rubin LL, Richardson WD (2004). Cooperation between sonic hedgehog and fibroblast growth factor/MAPK signalling pathways in neocortical precursors.. Development.

[12] Chandran S, Kato H, Gerreli D, Compston A, Svendsen CN (2003). FGF-dependent generation of oligodendrocytes by a hedgehog-independent pathway.. Development.

[13] Munoz-Sanjuan I, Brivanlou AH (2002). Neural induction, the default model and embryonic stem cells.. Nat Rev Neurosci.

[14] Miller RH, Dinsio K, Wang R, Geertman R, Maier CE (2004). Patterning of spinal cord oligodendrocyte development by dorsally derived BMP4.. J Neurosci Res.

[15] Mekki-Dauriac S, Agius E, Kan P, Cochard P (2002). Bone morphogenetic proteins negatively control oligodendrocyte precursor specification in the chick spinal cord.. Development.

[16] Attisano L, Tuen Lee-Hoeflich S (2001). The Smads.. Genome Biol.

[17] Massague J, Seoane J, Wotton D (2005). Smad transcription factors.. Genes Dev.

[18] Massague J, Gomis RR (2006). The logic of TGFbeta signaling.. FEBS Lett.

[19] Lu QR, Yuk D, Alberta JA, Zhu Z, Pawlitzky I (2000). Sonic hedgehog–regulated oligodendrocyte lineage genes encoding bHLH proteins in the mammalian central nervous system.. Neuron.

[20] Takebayashi H, Yoshida S, Sugimori M, Kosako H, Kominami R (2000). Dynamic expression of basic helix-loop-helix Olig family members: implication of Olig2 in neuron and oligodendrocyte differentiation and identification of a new member, Olig3.. Mech Dev.

[21] Tekki-Kessaris N, Woodruff R, Hall AC, Gaffield W, Kimura S (2001). Hedgehog-dependent oligodendrocyte lineage specification in the telencephalon.. Development.

[22] Schmiesing JA, Gregson HC, Zhou S, Yokomori K (2000). A human condensin complex containing hCAP-C-hCAP-E and CNAP1, a homolog of Xenopus XCAP-D2, colocalizes with phosphorylated histone H3 during the early stage of mitotic chromosome condensation.. Mol Cell Biol.

[23] Woodruff RH, Tekki-Kessaris N, Stiles CD, Rowitch DH, Richardson WD (2001). Oligodendrocyte development in the spinal cord and telencephalon: common themes and new perspectives.. Int J Dev Neurosci.

[24] Pera EM, Ikeda A, Eivers E, De Robertis EM (2003). Integration of IGF, FGF, and anti-BMP signals via Smad1 phosphorylation in neural induction.. Genes Dev.

[25] Kretzschmar M, Doody J, Massague J (1997). Opposing BMP and EGF signalling pathways converge on the TGF-beta family mediator Smad1.. Nature.

[26] Sapkota G, Alarcon C, Spagnoli FM, Brivanlou AH, Massague J (2007). Balancing BMP signaling through integrated inputs into the Smad1 linker.. Mol Cell.

[27] Tsang M, Dawid IB (2004). Promotion and attenuation of FGF signaling through the Ras-MAPK pathway.. Sci STKE.

[28] Warf BC, Fok-Seang J, Miller RH (1991). Evidence for the ventral origin of oligodendrocyte precursors in the rat spinal cord.. J Neurosci.

[29] Kuroda H, Fuentealba L, Ikeda A, Reversade B, De Robertis EM (2005). Default neural induction: neuralization of dissociated Xenopus cells is mediated by Ras/MAPK activation.. Genes Dev.

[30] Zhou Q, Wang S, Anderson DJ (2000). Identification of a novel family of oligodendrocyte lineage-specific basic helix-loop-helix transcription factors.. Neuron.

[31] Zhou Q, Choi G, Anderson DJ (2001). The bHLH transcription factor Olig2 promotes oligodendrocyte differentiation in collaboration with Nkx2.2.. Neuron.

[32] Xian HQ, Werth K, Gottlieb DI (2005). Promoter analysis in ES cell-derived neural cells.. Biochem Biophys Res Commun.

[33] Ying QL, Nichols J, Chambers I, Smith A (2003). BMP induction of Id proteins suppresses differentiation and sustains embryonic stem cell self-renewal in collaboration with STAT3.. Cell.

[34] Wichterle H, Lieberam I, Porter JA, Jessell TM (2002). Directed differentiation of embryonic stem cells into motor neurons.. Cell.

[35] Bouhon IA, Joannides A, Kato H, Chandran S, Allen ND (2006). Embryonic stem cell-derived neural progenitors display temporal restriction to neural patterning.. Stem Cells.

[36] Quandt K, Frech K, Karas H, Wingender E, Werner T (1995). MatInd and MatInspector: new fast and versatile tools for detection of consensus matches in nucleotide sequence data.. Nucleic Acids Res.

[37] Loots GG, Ovcharenko I (2004). rVISTA 2.0: evolutionary analysis of transcription factor binding sites.. Nucleic Acids Res.

[38] Kessaris N, Fogarty M, Iannarelli P, Grist M, Wegner M (2006). Competing waves of oligodendrocytes in the forebrain and postnatal elimination of an embryonic lineage.. Nat Neurosci.

[39] Kretzschmar M, Liu F, Hata A, Doody J, Massague J (1997). The TGF-beta family mediator Smad1 is phosphorylated directly and activated functionally by the BMP receptor kinase.. Genes Dev.

[40] Spagnoli FM, Hemmati-Brivanlou A (2006). Guiding embryonic stem cells towards differentiation: lessons from molecular embryology.. Curr Opin Genet Dev.

[41] Loebel DA, Watson CM, De Young RA, Tam PP (2003). Lineage choice and differentiation in mouse embryos and embryonic stem cells.. Dev Biol.

[42] Stavridis MP, Smith AG (2003). Neural differentiation of mouse embryonic stem cells.. Biochem Soc Trans.

[43] Keller G (2005). Embryonic stem cell differentiation: emergence of a new era in biology and medicine.. Genes Dev.

[44] Colak D, Mori T, Brill MS, Pfeifer A, Falk S (2008). Adult neurogenesis requires Smad4-mediated bone morphogenic protein signaling in stem cells.. J Neurosci.

[45] Murtaugh LC, Zeng L, Chyung JH, Lassar AB (2001). The chick transcriptional repressor Nkx3.2 acts downstream of Shh to promote BMP-dependent axial chondrogenesis.. Dev Cell.

[46] Kim DW, Lassar AB (2003). Smad-dependent recruitment of a histone deacetylase/Sin3A complex modulates the bone morphogenetic protein-dependent transcriptional repressor activity of Nkx3.2.. Mol Cell Biol.

